# Development and testing of a dual-frequency real-time hardware feedback system for the hard X-ray nanoprobe beamline of the SSRF

**DOI:** 10.1107/S1600577524010208

**Published:** 2025-01-01

**Authors:** Zhisen Jiang, Hui Jiang, Yinghua He, Yan He, Dongxu Liang, Huaina Yu, Aiguo Li, Riccardo Signorato

**Affiliations:** a Shanghai Synchrotron Radiation Facility Shanghai Advanced Research Institute, Chinese Academy of Sciences 239 Zhangheng Road, Pudong District Shanghai201204 People’s Republic of China; b Shanghai Institute of Applied Physics Chinese Academy of Sciences 2019 Jialuo Road, Jiading District Shanghai201800 People’s Republic of China; c S.RI.Tech, Viale Del Lavoro 42A, 35010Vigonza, Italy; Bhabha Atomic Research Centre, India

**Keywords:** X-ray, feedback, PID, FPGA, frequency domain

## Abstract

A novel dual-frequency real-time feedback system has been developed to simultaneously optimize and stabilize beam position and energy at the hard X-ray nanoprobe beamline of the Shanghai Synchrotron Radiation Facility. A user-selected cut-off frequency is used to separate the beam position signal obtained from an X-ray beam position monitor into two parts, *i.e.* high-frequency and low-frequency components. They can be real-time corrected and optimized by two different optical components, one chromatic and the other achromatic, of very different inertial mass, such as Bragg monochromator dispersive elements and a pre-focusing total external reflection mirror. The experimental results shown in this article demonstrate a significant improvement in position and energy stabilities. The long-term beam angular stability clearly improved from 2.21 to 0.92 µrad RMS in the horizontal direction and from 0.72 to 0.10 µrad RMS in the vertical direction.

## Abstract 2

We introduce a novel approach for a real-time dual-frequency feedback system which has been firstly used at the hard X-ray nanoprobe beamline of Shanghai Synchrotron Radiation Facility. It can efficiently stabilize the X-ray beam position and stability in parallel, making use of different optical systems in the beamline.

## 1. Introduction

In the past two decades both synchrotron light sources and X-ray focusing optics have developed significantly (Mino *et al.*, 2018 ▸). These advances allow X-rays to be focused further down to the nanoscale (Chayanun *et al.*, 2019 ▸). Nanofocusing-based X-ray characterization techniques are powerful tools that provide new research opportunities in a wide range of scientific fields such as biology and materials science, where they can provide valuable insights (Deng *et al.*, 2015 ▸; Yan *et al.*, 2016 ▸). This has led to the construction of hard X-ray nano­probe beamlines at various synchrotron radiation facilities (Johansson *et al.*, 2021 ▸; Martínez-Criado *et al.*, 2016 ▸; Quinn *et al.*, 2021*a* ▸).

Position instability and energy drifts of the X-ray beam are key factors in limiting the performance of a nanoprobe beamline. Positional and energy instability of the beam at the sample are caused by several factors such as unstable electron beam orbit, repeated injection of electron bunches into the storage ring, changes in the parameters of the insertion devices (Tsumaki & Kumagai, 2001 ▸), ground vibrations (Wang *et al.*, 2012 ▸), cooling of optics, thermal expansion (Yan *et al.*, 2017 ▸) or contraction of optical elements (Owen *et al.*, 2016 ▸), and nearby human activities. In real life, combinations of the aforementioned perturbations are known to seriously impact the stability of the beam position and of the selected energy as well as its coherence (Grizolli *et al.*, 2019 ▸), size, divergence (Goto, 2015 ▸) and flux, which all significantly affect the focusing and wavelength selection performance of the X-ray optics, therefore introducing artifacts in measurement accuracy and data quality of various experiments (Tian *et al.*, 2022 ▸). Hence, it is of paramount importance to minimize all beam instabilities for nanofocusing beamlines.

The concept and name for beamline intensity feedback were first introduced by Krolzig *et al.* (1984 ▸). After that, various beam stabilization schemes were developed and implemented to address specific challenges and requirements at different facilities. Nowadays, beam feedback systems have found successful applications in various synchrotron radiation experiments and play a critical role in improving beam stability, reducing beam jitter and significantly enhancing the overall performance of beamlines. By adjusting the incidence angles of optical elements (*e.g.* mirrors or monochromators) with real-time feedback control algorithms based on PID (proportional integral derivative) schemes, beam properties can be effectively corrected and optimized, using as input measured position and intensity information acquired from accurate and fast beam position monitors (BPMs). Recent developments in beam feedback systems based on X-ray BPMs and optics are focusing on improving the input measurement accuracy, its resolution and speed, and also optimizing control algorithms, even integrating advanced technologies such as machine learning for enhanced beam control and stability.

At Diamond Light Source in Oxfordshire, UK, a software-based feedback system was implemented to achieve intensity stability of the beam through modulation of the double-crystal monochromator (DCM) (Bloomer *et al.*, 2013 ▸). To reduce the influence of thermal drift and vibrations of multiple optical elements, a combination of a robot scheme and an active correction method were introduced, which successfully stabilized the beam (Quinn *et al.*, 2021*b* ▸). At the MX2 beamline of the Australian Synchrotron, the position of visible light produced by a neodymium-doped yttrium aluminium garnet (YAG) crystal was used to adjust the Kirkpatrick–Baez (KB) mirrors (Aragão *et al.*, 2018 ▸). For X-ray absorption spectroscopy (XAS) experiments at BL08U1A of the SSRF, a detailed description of an online feedback system that effectively maintained the optimal photon flux was demonstrated (Zhang *et al.*, 2023 ▸). The feedback systems at the Advanced Photon Source used the BPM and a feed-forward approach to adjust the beam angle by monochromator steering which maintained the beam stability at less than ±25 µm over an XAS scan (Fischetti *et al.*, 2004 ▸). Feedback systems are also used to achieve fast automated retuning of energy (Stepanov *et al.*, 2022 ▸).

Orbital stability, including both electron beam position and angle stability, directly affects the stability of the emitted photon beam. Therefore, using a feedback system to correct the orbit of the storage ring could also be an effective way to stabilize the X-ray beam. At the National Synchrotron Light Source II, beam position was corrected using three sequential feedback loops, including electron beam orbit, the DCM and the KB mirror feedback systems (Schneider *et al.*, 2021 ▸). At Synchrotron SOLEIL there is an orbit feedback system that performs well in the short to long term against environmental perturbations (Hubert & Cassinari, 2013 ▸; Engblom *et al.*, 2017 ▸). Recently, machine learning has been applied to source stabilization, achieving a source size stability of 0.2 µm (0.4%) at the Advanced Light Source (Leemann *et al.*, 2019 ▸).

The hard X-ray nanoprobe beamline (BL13U) of the Shanghai Synchrotron Radiation Facility (SSRF) was designed (He *et al.*, 2024 ▸; Li *et al.*, 2017 ▸) in 2016 and completed the beamline acceptance test in 2023. In order to achieve nanofocusing of X-rays, BL13U has a very long length, beyond 130 m. The resulting long lever arms make it rather difficult to achieve precise stabilization of beam properties at frequencies greater than 1 Hz.

The pitch (Bragg) angles of the double-crystal/multilayer monochromator directly determine energy selection and therefore its stability. Hence, it is not appropriate to use only monochromator steering to compensate for large low-frequency position drift of the X-ray beam, though the small mass of the Si crystals or, to a lesser extent, of the multilayer mirrors makes it possible to operate them at high PID frequency (*i.e.* up to 100 Hz and above). On the contrary, the large mass of a long achromatic focusing mirror leads to large inertia, which makes it impossible to operate a PID feedback at high frequency (*i.e.* at more than ∼10 Hz) to compensate for high-frequency vibrations.

Therefore, we proposed, developed and tested an innovative dual-frequency approach to PID feedback using a single position signal from an X-ray BPM to simultaneously fine-tune the mirror and the monochromator of the beamline at different frequencies. This article provides a detailed description of the dual-frequency feedback system as currently deployed at BL13U, including its composition, algorithm logic, software function, optimization and debugging.

## 2. Beamline layout

The BL13U optical design is based on a dual-stage focusing scheme. The first stage, known as pre-focusing, involves the use of a single bent mirror to focus the X-rays into a secondary source aperture (SSA). The second stage uses KB focusing mirrors to achieve 2D nanofocusing at the experimental station by re-imaging the SSA onto the sample. All optical elements of the beamline, except the vertically focusing mirror of the nanofocusing KB, deflect the beam in the horizontal plane in order to reduce as much as possible the impact of ground vibrations and gravity on position and quality of the beam spot. The two-stage focusing approach allows for precise control over photon flux, coherence length and focused spot size, mitigating issues due to imperfect beamline optics (de Jonge *et al.*, 2014 ▸) and vibrations. At BL13U, the position signal from a diamond-based BPM located immediately upstream of the SSA is used to monitor the stability of X-ray beams and fine-tune the pre-focusing mirror and monochromators, providing an as highly stable as possible source point to the downstream KB focusing optics.

**Figure 1:**
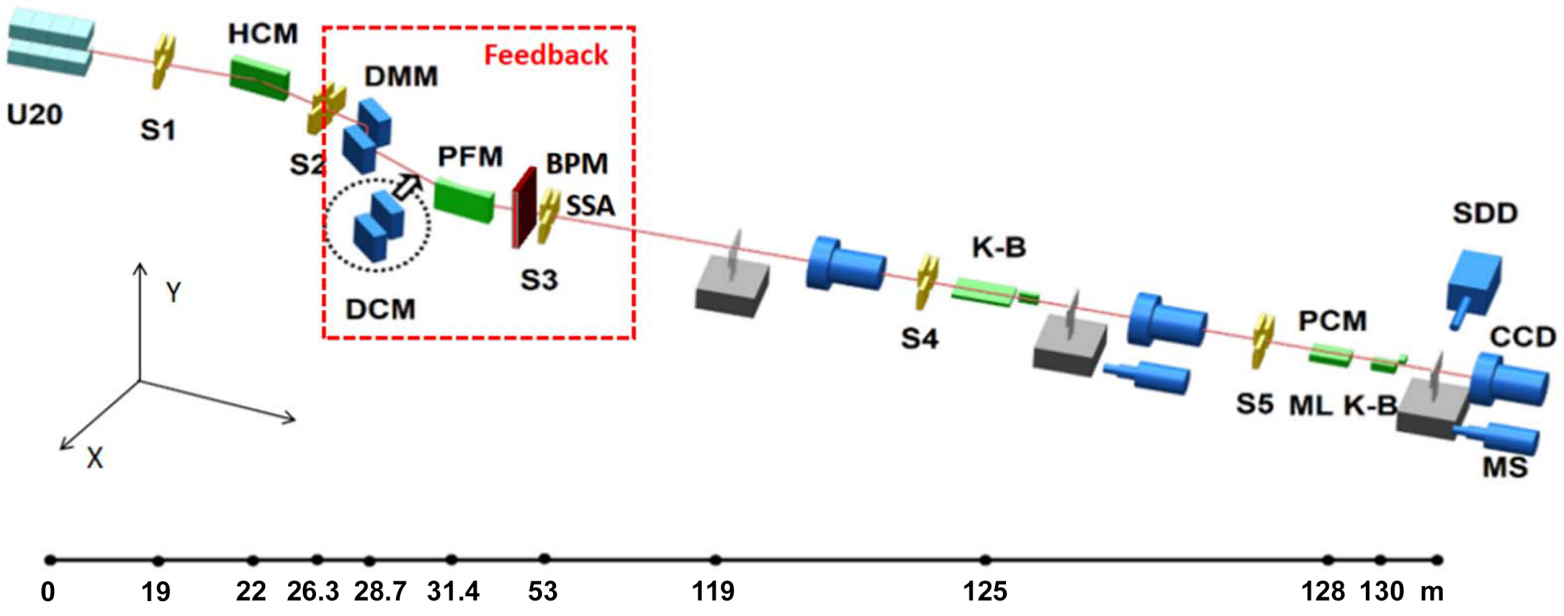
Beamline layout of the hard X-ray nanoprobe beamline at the SSRF.

The optical scheme upstream of the secondary source aperture, as can be seen in Fig. 1 ▸, consists of a bendable collimating mirror (HCM; 22.5 m), a staggered combination of double-multilayer monochromator (DMM; 26.3 m) and DCM (28.7 m), and a pre-focusing mirror with fixed toroidal shape (PFM; 31.4 m). These optical components are all horizontal deflecting. The HCM, with two stripes of metallic coatings, rhodium and platinum, has an effective acceptance angle of 70 µrad × 30 µrad with respect to the U20 undulator source of the beamline and is used to minimize the beam divergence before the monochromators. The DMM and DCM are alternatively used to fit different experimental requirements. Specifically, the high-flux fluorescence mode employs the wide energy bandpass of the DMM, while the high-energy-resolution mode of the beamline makes use of the DCM. This flexibility allows for a broad range of different experimental requirements and techniques to be satisfied. The DMM has two multilayer stripes covering different energy ranges: (i) Ru/C with 3.1 nm period for the energy range 8.3–25 keV and (ii) Ni/C with 3.5 nm period for the energy range 5–8.3 keV. The energy bandpass is Δ*E*/*E* ≃ 10^−2^ and the dimensions of both multilayer mirror substrates are 230 mm × 35 mm × 40 mm and their weights are ∼0.75 kg. The DCM, employing a Si 〈111〉 crystals set, operates within an angular range of 4–24°. This allows an energy range of 5–25 keV to be covered, providing high-resolution beam (Δ*E*/*E* ≃ 10^−4^) for various experimental needs. The dimensions of the crystals are 50 mm × 50 mm × 20 mm (first crystal) and 100 mm × 30 mm × 20 mm (second crystal) with weight ∼0.14 kg. Both DMM and DCM are cooled by a closed-loop liquid-nitro­gen cryocooler.

Then, the PFM focuses the beam both horizontally and vertically onto the SSA. The PFM, coated with rhodium, operates at a fixed grazing incidence angle of 2.5 mrad. The dimensions of the Si mirror substrate are 700 mm × 50 mm × 50 mm and its weight is ∼4.08 kg. The size of the SSA (S3) can be defined by tunable vertical and horizontal slits, in order to be adjusted to meet the requirements of either high photon flux or selection of full coherence for diffraction-limited nanofocusing. Finally, a total-reflection KB nanofocusing system at 125 m and a multilayer KB nanofocusing system at 130 m from the source are capable of achieving two-dimensional focal spot sizes of below 50 nm × 50 nm and 26 nm × 17 nm (Jiang *et al.*, 2024 ▸) (FWHM), respectively.

## 3. Beam monitoring approach

A CVD diamond quadrant BPM with thickness of 150 µm and read through a fast digital picoammeter (TetrAMM) is placed directly in front of the SSA of the beamline, at about 52.5 m from the source. It is used for simultaneously recording intensity, horizontal and vertical X-ray beam positions with high accuracy and a large bandwidth of up to 5 kHz. The electric current signals from each quadrant of the BPM, read and digitized by the fast picoammeter located next to the detector, are first properly interpolated to derive actual beam position information, and then filtered to be split into their high-frequency and low-frequency parts. The equations used to derive the X-ray beam position in a 45° rotated BPM geometry are the following,
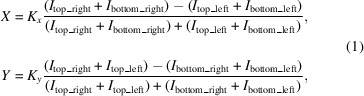
where *I* with the position index is the electric current signal read by each of the four quadrants of the BPM, and *K*_*x*_ and *K*_*y*_ are scaling factors.

**Figure 2:**
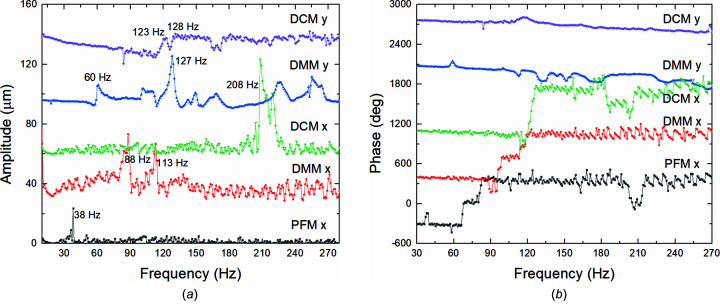
Bode diagram of the pitch angle of the DMM, DCM and PFM: (*a*) amplitude and (*b*) phase plots.

When switching between the DCM and the DMM, their pitch and roll piezo actuators of the second crystal/multilayer can be used to compensate for the high-frequency parts of the X-ray beam drifts and vibrations in real time. Instead, the PFM is used for compensation feedback of the low-frequency part, with a user-selectable upper actuation frequency. We first characterized the first eigenfrequencies of the DMM and DCM optics as well as of the PFM by a frequency analysis tool and they are, respectively, 88 Hz, 208 Hz and 38 Hz in the horizontal direction. The responses to sinusoidal waves applied to the piezos with fixed amplitude and sweeping up to ∼300 Hz frequency bandwidth were measured. The measurements were carried out with the beam and the cooling on. The Bode diagram can be found in Fig. 2 ▸. *X* is the horizontal and *Y* is the vertical direction.

In the context of a nanoprobe beamline, a mere 1.5% RMS fluctuation in flux can have a significant impact on the test accuracy of the focus spot size and degenerate coherent illumination conditions. Based on the optical layout of BL13U, the natural focal spot at the secondary source aperture (SSA) is 452 µm × 26 µm FWHM, and the SSA is used to shrink it to a size of 46 µm × 11 µm FWHM. Flux stability can therefore be interpreted as the relative motion of the focused beam with respect to the SSA openings in the horizontal (*X*) and vertical directions (*Y*), respectively. The theoretical allowable maximum beam displacement on the SSA is 21.6 µm in the horizontal direction and 1.65 µm in the vertical direction when maximum beam stability and beam flux is required.

Factors that significantly influence the spot size in the vertical direction include: photon source size and stability, as well as displacement, roll and pitch angles of HCM, DCM/DMM and PFM. By adjusting the optics angles, the beam position at the BPM can be corrected. The beam position deviation at the BPM, Δ*X* and Δ*Y*, can be analytically expressed as (Sergueev *et al.*, 2016 ▸)
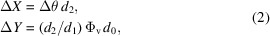
where Δθ is the vibration of the grazing incidence angle of the optics, Φ_v_ = 2sinΔφsinθ_m_ is the deviation angle in the vertical direction, Δφ is the vibration of the roll angle, θ_m_ is the grazing incidence angle of the monochromator, *d*_0_ is the distance between the source and the monochromator, *d*_1_ = 31.4 m is the distance between the source and PFM, and *d*_2_ = 21.1 m is the distance between the PFM and the BPM. Based on equation (2), the maximum acceptable stability of monochromators and PFM can build a relationship with the allowable maximum beam offset of the SSA of 21.6 µm × 1.65 µm in order to ensure that the stability of the beam intensity is better than 1.5%, with 90% of the allowable offset error component allocated to the vertical direction and 10% allocated to the horizontal direction. The angular stability of each optical element can be estimated by measuring the beam offset at the BPM.

## 4. Dual-frequency feedback system design and implementation at BL13U

The new, dual-frequency, beamline enhanced stabilization technology system, hereinafter called BiBEST, is a software and instrumentation suite especially conceived to simultaneously control and stabilize the position (horizontal and vertical) and intensity of the photon beam in synchrotron or X-ray free-electron laser beamlines. It is based on the established commercially available BEST system hardware, manufactured by CAEN ELS Srl (https://www.caenels.com/product/best/). It was designed and developed following a specific initial request from SSRF by S.RI.Tech Srl (official distributor for BiBEST), with specific software support from CAEN ELS staff. The software is commercially available through S.RI.Tech Srl (Sri_tech@pec.it).

With respect to previous standard feedback systems, it allows a selective, parallel and frequency-tunable control and stabilization of multiple actuators acting on different bandwidths of the same input BPM signal. This approach is very well suited to beamlines where a mix of fast and slow actuators operating on the same beam property are installed. A typical example is simultaneous control of heavy and therefore slow achromatic optical elements (*i.e.* BL13U PFM) coupled with lightweight chromatic ones (*i.e.* BL13U monochromator crystals and multilayer mirrors). The BiBEST algorithms separate the input X-ray BPM signals components higher than and lower than a given cut-off frequency, and then feed them into two separated PID correction loops operated synchronously. The low-frequency part is then applied to the slow PFM whilst the high-frequency part goes to either one of the monochromators, depending on which one is in use. Clearly, the PFM and both monochromators do include piezoelectric-driven pitch control.

**Figure 3:**
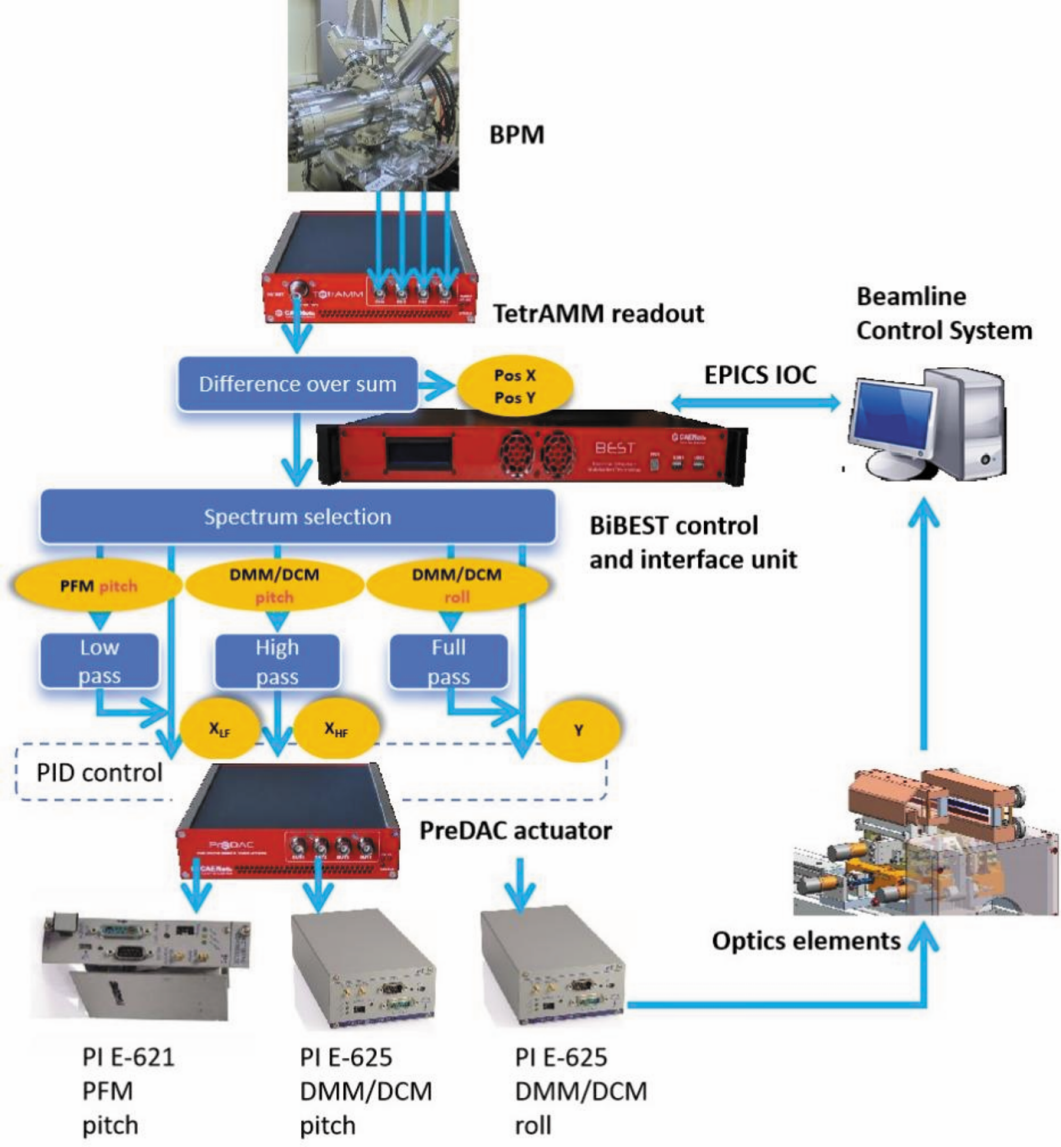
Logic diagram of a typical implementation of the BiBEST system.

As can be seen in Fig. 3 ▸, three hardware instrumentation building blocks are included in the BiBEST suite: (i) the readout block, *i.e.* a CAEN ELS TetrAMM fast digital picoammeter, (ii) the control and interface block, *i.e.* a BiBEST control and interface central unit, and (iii) the actuator block, *i.e.* a CAEN ELS PreDAC digital DAC unit.

The first building block of the BiBEST system is a current readout device called TetrAMM, which is a fast digital picoammeter characterized by high sampling speed (up to 100 kHz), directly connected to the outputs from an X-ray BPM and located as close to the sensor as reasonably possible (typically ∼1 m). The average noise for the four channels of the picoammeter was 0.457 ± 0.002 nA. The currents from the four-quadrant BPM are acquired, immediately digitalized and sent from the TetrAMM to the BiBEST control and interface central unit via a direct optics fiber link. The BiBEST control and interface unit performs all calculations required to obtain beam position and intensity information from the raw BPM data and splits it within the required frequency bandwidths. Then it calculates the corrections necessary to stabilize the position and energy of the beam at the desired setpoint using a fast PID algorithm. The correction setpoints are finally sent, also via a direct optics fiber link, to a PreDAC unit that uses its internal high-precision digital-to-analog converters to generate an output voltage signal capable of driving piezoelectric actuators acting on the optical elements. Also the PreDAC unit is located as close to the sensor as reasonably possible (typically ∼1 m).

**Figure 4:**
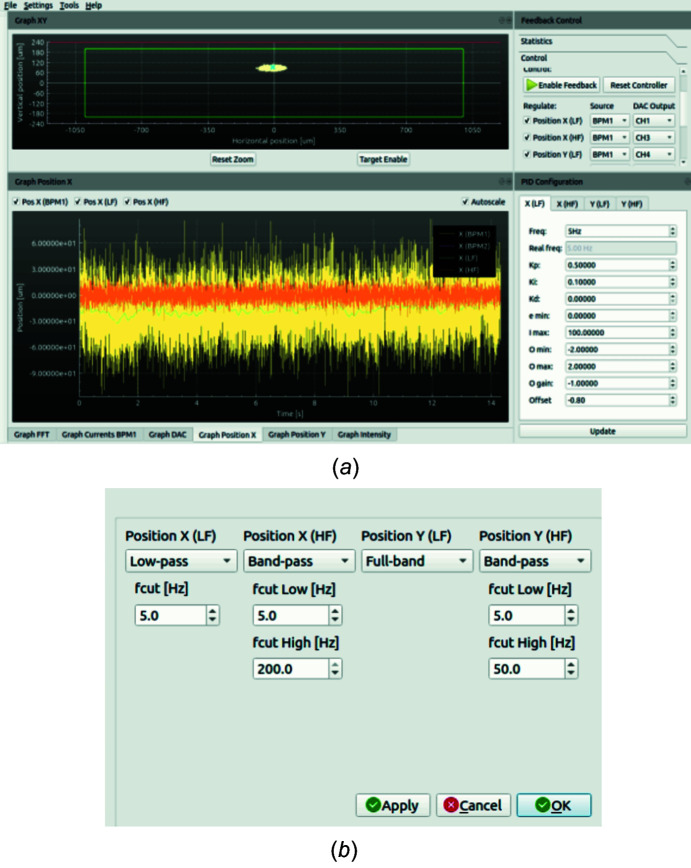
(*a*) Screenshot of the local GUI, which allows the BL operator to monitor, manage and control beam position and intensity. The lower right corner shows the setting of PID parameters in use. (*b*) The frequency selection and filter setting interface for the four PID channels available. X is horizontal and Y is vertical.

The critical task of real-time PID calculations is performed directly in dedicated FPGA hardware to insure a fully deterministic computing time, maximum calculation speed and minimum elaboration delay. The FPGA executes also the control PID algorithms in an optimized way, adding a very low delay to the feedback loop in order to guarantee the BiBEST correction and stabilization performances over the highest possible frequency spectrum. The X-ray beam intensity is also constantly monitored by the BiBEST and can be used to automatically enable or disable the PID controller in case the X-ray beam is shut down (beam loss) or blocked by a shutter. The control and interface unit includes a local graphical interface, named Local GUI, shown in Fig. 4 ▸(*a*), which allows the X-ray beam position and intensity to be fully monitored, managed and controlled. A standard 10/100/1000 TCP-IP Ethernet link allows remote control and configuration of this system; hence it is possible to connect the BiBEST control unit directly to the beamline control system. The BiBEST also provides several tools to monitor and analyze the X-ray beam in real and frequency space, allowing the effect of its PID feedback to be analyzed and fine-tuned in real time.

The feedback actuation frequency is a parameter of paramount importance and is strictly correlated to and fully determined by the dynamical performances of the positioning mechanics driven by the PID. Certain positioners are very stiff, lightweight and fully backlash-free, and therefore can be actuated to track the setpoints generated by the PID at relatively high frequencies of up to even a few hundreds of Hz. Other positioners may be intrinsically slower, due to for example the sheer mass of the optical element that they position, or less stiff by design or also exhibit large backlash. Therefore, they can be actuated at frequencies of only a very few tens of Hz at most. As seen in Fig. 4 ▸(*b*), the BiBEST allows setting different feedback actuation frequencies. Slow beam position drifts, usually due to temperature changes, are easily compensated by running the PID at just around 1 Hz whilst seismic or cooling-related vibrations compensations requires PID frequencies of ideally 100 Hz or even above. Table 1 ▸ reports the specific arrangement of feedback channels as implemented for the BL13U beamline.

When the feedback is on, the PFM pitch (low-frequency part of the BPM horizontal position signal), as well as pitch (high-frequency part of the BPM horizontal position signal) and roll (unfiltered, full-band part of the BPM vertical position signal) of the second crystal/mirror of either DCM/DMM can all be controlled simultaneously and in a synchronized way.

## 5. Experimental results

Hereafter we report experimental data collected during the initial commissioning of the BL13U beamline, when we installed and characterized the first, prototype, BiBEST unit. An as extensive as possible measurements campaign was carried out within the available beam time, allowing functionality and performance of the new feedback unit to be assessed. We describe in the following paragraphs all undertaken activities and measurements.

**Figure 5:**
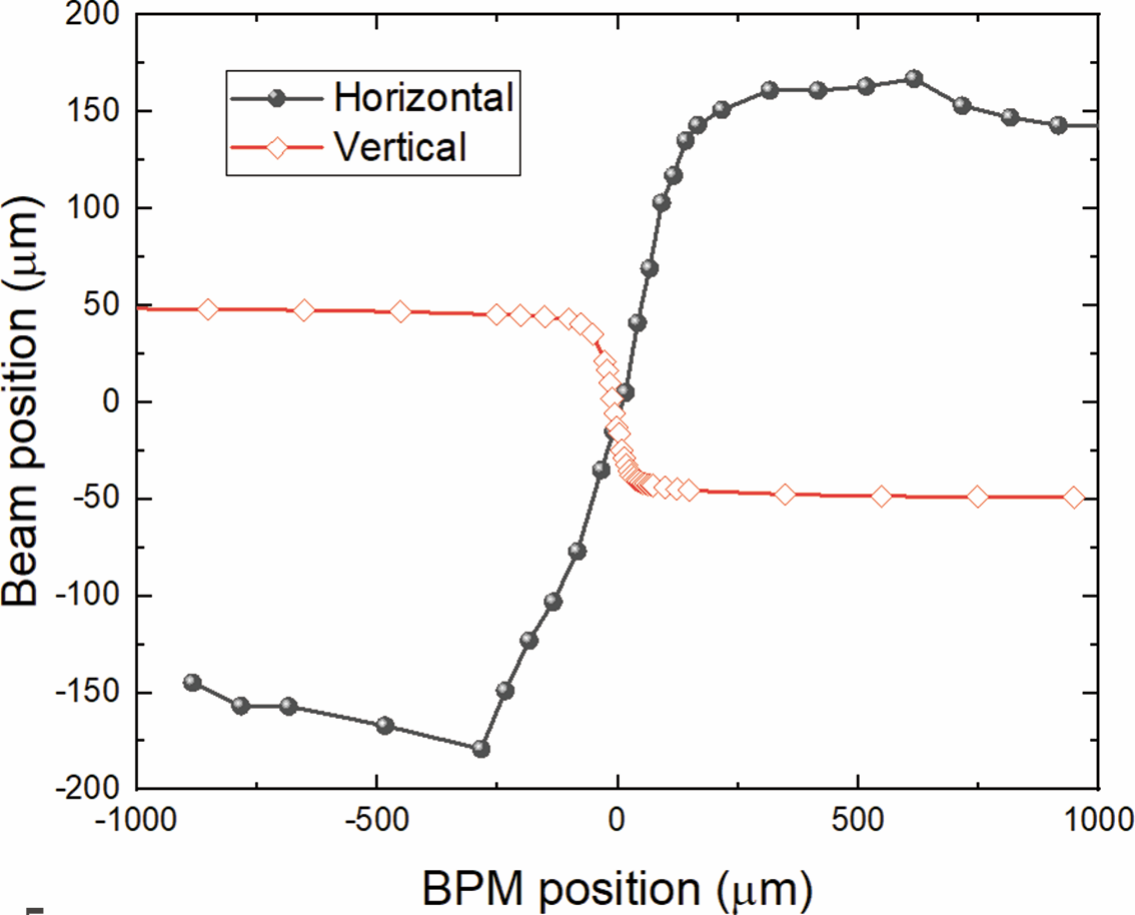
Determination of the linear region of the diamond-based X-ray BPM.

First, aligning the X-ray beam to the center of the diamond BPM, which is its linear region as can be seen in Fig. 5 ▸, is a crucial step. The diamond detector is linear in an area that is roughly comparable with the size of the X-ray spot: ∼400 µm × 80 µm. Only when the spot is located in this area is the beam position calculated based on the four-channel currents accurate enough to be used as input for real-time PID and correction using the BiBEST, which samples the detector signal at 5 kHz.

Most of the tests in this article were carried out when the beamline was run in high-flux fluorescence mode using the DMM. As shown in the upper right of Fig. 4 ▸(*a*), three PreDAC channels (the checked CH1, CH3 and CH4) were used for angular feedback of the X-ray optics. Subsequently, we also acquired some short-term stability data in high-energy resolution mode by using the DCM. In this mode, the original CH3 was changed to CH2 and CH4 was connected to the DCM second crystal roll.

**Figure 6:**
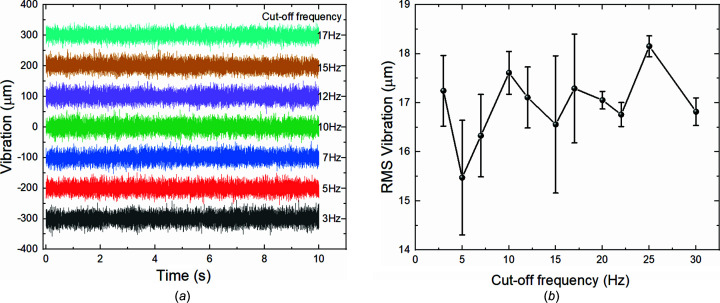
Measured vibration data sampled at 1 kHz over 10 s in the horizontal direction. (*a*) Comparison of raw data using different cut-off frequencies, and (*b*) comparison of RMS vibration levels associated with the same cut-off frequencies.

Short-term beam stability tests were performed with a sampling interval of 1 ms over 10 s, hence covering the 0.1 to 500 Hz bandwidth. Fig. 6 ▸(*a*) shows plots of the measured stability data using different cut-off frequencies for the low-frequency PID in the horizontal direction and Fig. 6 ▸(*b*) compares the related RMS vibration deviation while the beamline was operated in high-flux fluorescence mode. It can be seen that, for the dual-band cut-off frequency of 5 Hz, the RMS vibration level at the SSA is minimized.

**Figure 7:**
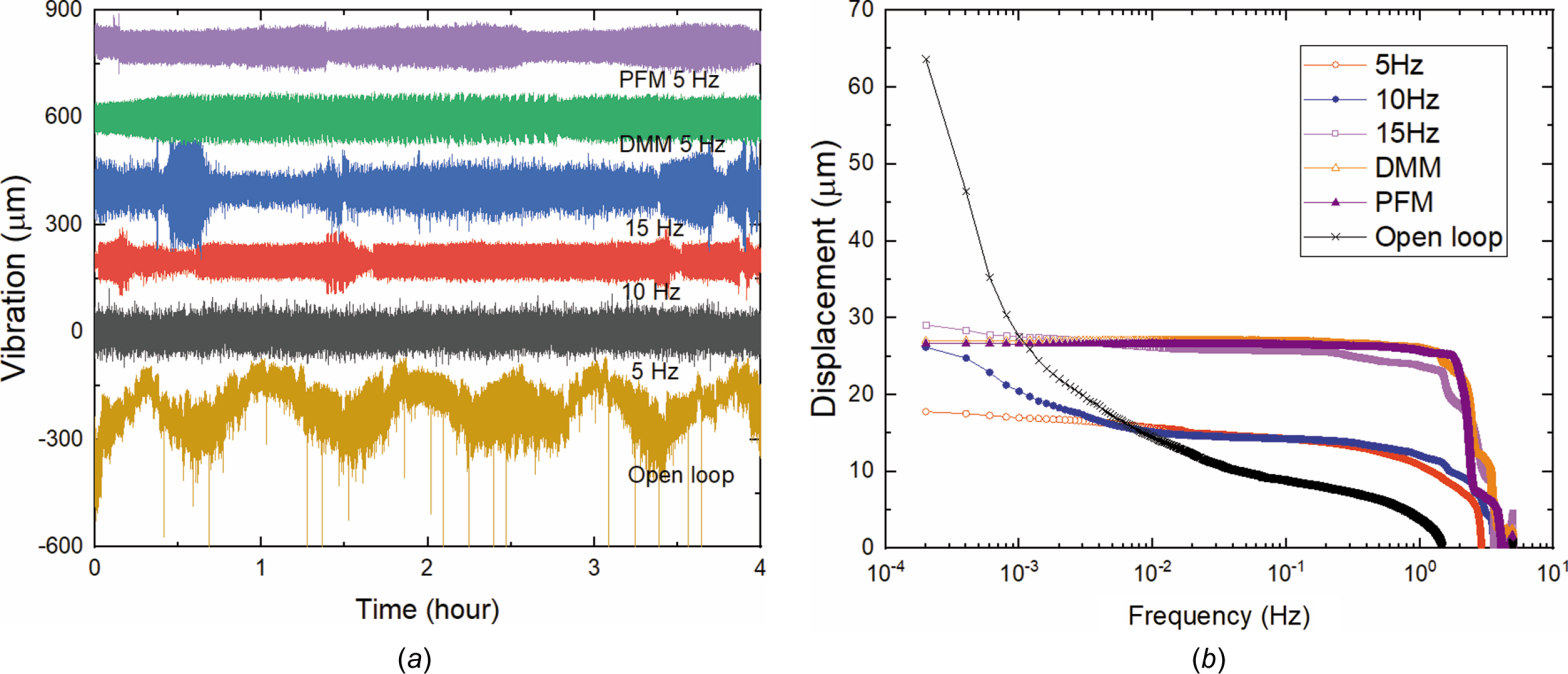
(*a*) Comparison of measured vibration data in the horizontal direction under different cut-off frequencies and (*b*) cumulative vibrations in the frequency spectrum.

The following Fig. 7 ▸(*a*) compares long-term measured vibration data in the horizontal direction collected with sampling interval of 0.1 s under open-loop and dual-frequency closed-loop conditions over 4 h. The beamline in open-loop mode exhibits strong low-frequency oscillations with a time period of approximately one hour, whilst the beam position at the BPM under closed-loop condition remains very stable. Some bumps occur in the plots due to the sudden beam deviation from source or upstream optics. The cumulative spectral power of vibration displacements shown in Fig. 7 ▸(*b*) reveals that, at the low-frequency regime, the vibration amplitude with dual-frequency feedback on was always sensibly smaller than in open loop.

In our experiments, the long-term angular stability of the closed-loop feedback beam was clearly improved compared with the open-loop situation, respectively decreasing from 2.21 ± 0.37 to 0.92 ± 0.13 µrad RMS in the horizontal direction and decreasing from 0.72 ± 0.11 to 0.10 ± 0.02 µrad RMS in the vertical direction. We systematically changed the cut-off frequency from 5 to 10 and 15 Hz to check its influence on the vibration level. The results reveal that, if the cut-off frequency was set too high, such as more than 10 Hz, most of the vibrations in the frequency span cannot be efficiently suppressed and may be even amplified, despite the fact that the feedback can always overcome low-frequency vibrations.

**Figure 8:**
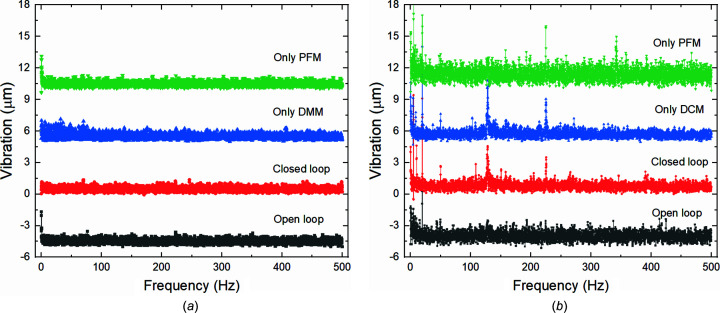
Short-term beam stability comparison using open loop, dual-frequency closed loop with a cut-off frequency of 5 Hz, and closed loops with single pitch adjustments by either DMM or PFM in the frequency domain working in (*a*) high-flux fluorescence mode (DMM) and (*b*) high-energy-resolution mode (DCM).

Then we also tested the use of feedback on only one component, *i.e.* the DMM or PFM. Fig. 7 ▸ shows that, due to the lack of partial frequency compensation, single-component feedback only improves the ultra-low-frequency stability of the beam but significantly amplifies the vibration higher than 0.01 Hz. Fig. 8 ▸(*a*) shows the frequency analysis of the measured stability data based on open-loop versus closed-loop feedback with the cut-off frequency of 5 Hz, and feedback using only the DMM or PFM. The RMS vibration data are presented in Table 2 ▸. Under short-term sampling, the high-frequency correction of the DMM can improve the beam stability to a certain extent. Dual-frequency feedback can reduce the RMS vibration from 19.8 µm to 15.5 µm. When the beamline was operated in high-energy-resolution mode with the DCM, the difference in short-term stability is even more pronounced. As can be seen in Fig. 8 ▸(*b*) and Table 2 ▸, the use of DCM or PFM alone amplifies fluctuations; especially for feedback cases using only the PFM, vibrations at almost all frequencies are amplified by a factor of approximately 1.5. When using the DCM for feedback, a significant vibration was generated at 128 Hz that can be compared with the resonance frequency of the DCM in the vertical direction.

**Figure 9:**
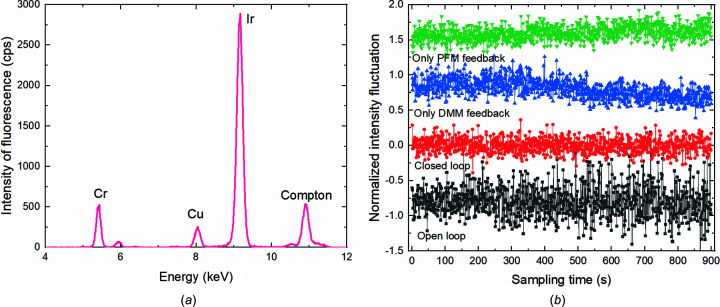
(*a*) Spectrum of the fluorescence signal. (*b*) Normalized fluorescence intensity fluctuation as a function of sampling time using open loop, dual-frequency closed loop with a cut-off frequency of 5 Hz, and closed loops with single pitch adjustments of either DMM or PFM.

One of the driving factors in our development of the BiBEST is to make spatial and spectral stability maximization possible fully in parallel, at the same time. The experimental results indicate that feedback from a single component may not necessarily be beneficial if the target is to stabilize both position and energy at the same time. The dual-frequency multi-actuator capabilities of the BiBEST are indeed required. We set the energy of the DMM monochromator to 11 keV, which happens to be the *L*_3_ absorption edge of iridium (Ir). At this energy, small energy fluctuations can cause significant fluctuations in the fluorescence signal of the Ir material [Fig. 9 ▸(*a*)] at the focus of the total-reflection KB nanofocusing system detected by a silicon drift detector (Vortex), and the energy stability can be compared semi-quantitatively using the incident photons intensity detected by a diamond BPM (Cividec B9) located just upstream of the sample as a normalization. As can be seen in Fig. 9 ▸(*b*) and Table 2 ▸, the results show that the fluctuation of Ir fluorescence intensity was significantly improved by a factor of two using the dual-frequency closed-loop feedback. While a single-frequency feedback on DMM or PFM can also improve fluorescence intensity fluctuations, there will be a low-frequency intensity drift over the sampling time, especially if the DMM is used alone.

## 6. Conclusion

In this article we introduce a novel approach for a real-time dual-frequency feedback system, which has been firstly used at the hard X-ray nanoprobe beamline of SSRF. The dual-frequency feedback system splits and simultaneously optimizes the high-frequency and low-frequency parts of vibrations and drift of the X-ray beam. It makes use of chromatic and achromatic optical components of different weights and sizes, thus effectively and simultaneously improving position and energy stability of the beam, fully cancelling vibrations at the low-frequency regime. Our long-term stability tests report a significant improvement in horizontal beam position and full correction of low-frequency vibrations. Energy stability, whilst keeping beam position locked, is also maximized. The BiBEST can then efficiently stabilize X-ray beam position and stability in parallel, making use of different optical systems in the beamline.

## Figures and Tables

**Table 1:** Detailed arrangement of feedback channels in the current implementation of the BiBEST system at BL13U

PreDAC output	Controlled device	Angular adjustment	Direction controlled
CH1	PFM incidence angle	Slow pitch	Horizontal
CH2	DCM second crystal pitch	Fast pitch	Horizontal
CH3	DMM second mirror pitch	Fast pitch	Horizontal
CH4	DMM/DCM second crystal/mirror roll	Slow/fast (full bandwidth) roll	Vertical

**Table 2:** Comparison of the vibration data (RMS)

	Open loop	Closed loop (5 Hz)	Only DMM	Only DCM	Only PFM
Short-term vibration (µm) in high-flux mode	19.8	15.5	19.1	/	20.4
Short-term vibration (µm) in high-resolution mode	41.1	37.3	/	44.3	62.5
Long-term vibration (µm)	55.4	22.7	34.4	/	29.9
Normalized fluorescence intensity fluctuation (%)	0.70	0.31	0.32	/	0.37
